# Chrysoeriol Improves In Vitro Porcine Embryo Development by Reducing Oxidative Stress and Autophagy

**DOI:** 10.3390/vetsci10020143

**Published:** 2023-02-10

**Authors:** Chao-Rui Wang, He-Wei Ji, Sheng-Yan He, Rong-Ping Liu, Xin-Qin Wang, Jing Wang, Chu-Man Huang, Yong-Nan Xu, Ying-Hua Li, Nam-Hyung Kim

**Affiliations:** Guangdong Provincial Key Laboratory of Large Animal Models for Biomedicine, School of Biotechnology and Health Sciences, Wuyi University, Jiangmen 529000, China

**Keywords:** chrysoeriol, embryo development, porcine, oxidative stress, autophagy

## Abstract

**Simple Summary:**

In this experiment, porcine embryos were used as a model, and the flavonoid chrysoeriol (CHE) was added to the in vitro culture of the porcine embryo. Then, the effect of CHE on embryo development was studied by microscopic observation, immunofluorescence staining, and quantitative Real-time PCR (qRT-PCR). The results showed that CHE could reduce the accumulation of reactive oxygen species (ROS) and the level of oxidative stress during the development of porcine embryos, thus improving the antioxidant activity of the embryos. We also found that CHE can increase the mitochondrial membrane potential in the embryos and protect mitochondrial function. Further experiments preliminarily showed that CHE can reduce apoptosis and autophagy during embryonic development. We have proved that CHE has good antioxidant activity, and this is the first time that CHE has been used in mammalian embryo culture in vitro. The results show that CHE can improve the development of pig embryos in vitro by reducing oxidative stress and autophagy.

**Abstract:**

Chrysoeriol (CHE) is a flavonoid substance that exists in many plants. It has various physiological and pharmacological effects, including anti-inflammatory, antioxidant, anti-tumor, and protective activity, especially for the cardiovascular system and liver. Among common livestock embryos, porcine embryos are often considered high-quality objects for studying the antioxidant mechanisms of oocytes. Because porcine embryos contain high levels of lipids, they are more vulnerable to external stimuli, which affect development. Our study explored the influence of CHE supplementation on oxidative stress in porcine oocytes and its possible mechanisms. Different concentrations of CHE (0, 0.1, 1, and 3 µM) were supplemented in the in vitro culture medium of the porcine oocytes. The results showed that supplementation with 1 µM CHE significantly increased the blastocyst rate and total cell number of embryos in vitro. After finding the beneficial effects of CHE, we measured reactive oxygen species (ROS), glutathione (GSH), and mitochondrial membrane potential (MMP) when the oocytes reached the 4-cell stage of development and determined the levels of apoptosis, cell proliferation, and autophagy at the blastocyst stage of development. The expression levels of some related genes were preliminarily detected by qRT-PCR. The results showed that the apoptosis of blastocysts in the CHE-treated culture also decreased compared with the untreated culture. Furthermore, CHE downregulated intracellular ROS and increased GSH in the embryos. CHE was also shown to improve the activity of mitochondria and inhibit the occurrence of autophagy. In addition, antioxidant-related genes (SOD1, SOD2, and CAT) and cell pluripotency-related genes (SOX2, OCT4, and NANOG) were upregulated. At the same time, apoptosis-related (Caspase 3) and autophagy-related (LC3B) genes showed a downward trend after supplementation with CHE. These results indicate that CHE improved the development of porcine embryos in vitro by reducing oxidative stress and autophagy levels.

## 1. Introduction

The in vitro culture (IVC) of embryos is a traditional technique widely used in the in vitro production (IVP) of animal embryos; in vitro culture includes the in vitro maturation (IVM) stage [1]. IVC has excellent potential for applications in artificial assisted reproduction technology (ART) and understanding of the biological basis [2,3,4]. In the past few decades, although some progress has been made in the field of IVP, the efficiency and quality of IVP are not as good as those of in vivo production because embryos are affected by the external environment and because of metabolic characteristics during growth and development [5,6,7]. The excessive accumulation of reactive oxygen species (ROS) is one of the causes of oxidative stress, which will seriously affect the development quality of embryos, especially in the process of in vitro culture [8]. The excessive accumulation of ROS can result in various types of damage to embryos and oocytes, including mitochondrial dysfunction [9], lipid peroxidation [10], apoptosis [10], and DNA damage [11]. Therefore, improving in vitro oocyte maturation and subsequent embryo development of animals is helpful by continuously studying compounds that exert antioxidant activity by reducing ROS levels [12].

Among common livestock embryos, porcine embryos contain more lipid droplets and the lipid will be transferred in the early stage of development, making the embryos more susceptible to ROS and oxidative stress [13,14]. Therefore, porcine oocytes are often used as a model for antioxidant research during embryonic culture in vitro. The addition of antioxidants at the early stage of porcine embryo development can reduce oxidative stress and improve developmental capacity. Multifarious antioxidants, such as melatonin [15,16], laminarin [17], imperatorin [18], oroxin A [19], and wedelolactone [20], have been reported to have antioxidant activity and can improve the early development ability of porcine embryos. However, the current IVC environment does not strongly imitate the environment in vivo. Therefore, it is important to further elucidate the mechanisms of antioxidant damage and to continue exploring effective antioxidants for oocyte culture to improve embryonic development in vitro [8].

Flavonoids are polyphenolic compounds that are abundant in nature and food and have functions related to diet and disease prevention [21]. Chrysoeriol (CHE), a flavonoid compound also known as 5,7- dihydroxy-2-(4-hydroxy-3-methoxyphenyl) chromen-4-one, is mainly distributed among plants, such as *Perilla frutescens* [22], *Phyllanthus species* [23], and *Salvia verticillata* [24] and the leaves of *Digitalis purpurea* (foxglove) [25]. According to previous reports, CHE has various physiological and pharmacological effects, including anti-inflammatory [25,26], antioxidant [24,27], anti-tumor [28,29], and cardiovascular and liver protective effects [30,31,32]. According to these studies, CHE showed good antioxidant activity. However, the impact of CHE on the reproductive system and oocytes has yet to be studied and reported.

We hypothesized that the addition of CHE in the IVC stage can improve the oocyte development quality of porcine embryos by decreasing oxidative stress. In this study, we first explored the effect of CHE on the development of porcine embryos. Then, some classic indexes related to oxidative stress (such as ROS, etc.) were detected. Changes in the apoptosis and autophagy levels of CHE during embryo development were also preliminarily evaluated. In this manner, the role of CHE in the development of porcine embryos is described.

## 2. Materials and Methods

### 2.1. Animals and Chemicals

All reagents used in this study were purchased from Sigma–Aldrich (St. Louis, MO, USA) unless otherwise indicated. All manipulations were performed on a heated stage set at 37.5 °C. Porcine ovaries used in this study were obtained from a slaughterhouse (Jiangmen, China).

### 2.2. Oocyte Collection and In Vitro Maturation (IVM)

We obtained the porcine ovaries from a local slaughterhouse, stored them in a thermos bottle filled with sterile saline solution supplemented with 100 µg/mL penicillin G, kept the ovaries at ~37 °C, and sent them to the laboratory within two to three hours. Next, the cumulus-oocyte complexes (COCs) were extracted from ovarian three-to-eight-mm antral follicles using a 10 mL syringe and an 18-gauge needle. Approximately 100 oocytes were placed in each well of four-well embryo culture plates (Nunc, Roskilde, Denmark) containing 500 µL of mineral oil-covered IVM (M199 medium with 10% porcine follicular fluid, 0.91 mM sodium pyruvate, 10 ng/mL of epidermal growth factor, 1 µg/mL insulin, 10 IU/mL of follicle-stimulating hormone, and 10 IU/mL of luteinizing hormone). The COCs were cultured at 38.5 °C in an atmosphere of 5% CO_2_ for 44 to 46 h.

### 2.3. Parthenogenetic Activation and Embryo IVC

After 45 h of IVM culture, COCs were transferred to 400 µL of 0.1% hyaluronidase for pipetting about 30 times, and then oocytes and granulosa cells were separated. The oocytes were selected and put into the activation solution (300 mM mannitol containing 0.5 mM HEPES, 0.05 mM CaCl2 2H2O, 0.1 mM MgSO4 7H2O, and 0.01% polyvinyl alcohol), followed by parthenogenetic activation using two direct-current (DC) pulses of 120 V for 60 µs. Next, the parthenogenetically activated oocytes were transferred to IVC medium containing 7.5 mg/mL cytochalasin B at 38.5 °C in an atmosphere of 5% CO_2_ for three hours, thereby inhibiting the discharge of the second polar body. Oocytes were washed four to five times with IVC after three hours of cytochalasin B treatment. About 40 to 50 oocytes were placed in each well of the four-well plate with 50 µL IVC medium with or without (0 µM, control group) CHE (TargetMol, Boston, MA, USA) that was finally dissolved with IVC at concentrations of 0, 0.1, 1, and 3 µM and then cultured at 38.5 °C in an atmosphere of 5% CO_2_ for seven days. On day 7, there were about 10–20 blastocysts in each group, and the experiment in each group was repeated at least three times.

### 2.4. Total Cell Number Count and Terminal Deoxynucleotidyl Transferase-Mediated dUTP Nick-End Labeling (TUNEL) Assay

Following the manufacturer’s instructions, apoptosis was analyzed using the TUNEL detection kit (Roche Diagnostics, IN, USA). In short, blastocysts formed on day 7 were collected and washed four times with phosphate-buffered saline (PBS) with 0.1% polyvinyl alcohol (PBS-PVA). The washed blastocysts were fixed in PBS-PVA containing 3.7% paraformaldehyde for 30 min and then permeabilized in 0.1% Triton X-100 for 30 min, all at room temperature. After permeabilization, the blastocysts were washed in a 96-well plate three to four times and fixed in blocking buffer (1% BSA in PBS-PVA) for one hour. Next, the blastocysts were incubated with fluorescein-conjugated dUTP and the terminal deoxynucleotidyl transferase enzyme (Roche) in the dark at 37.5 °C for one hour. Subsequently, the blastocysts were incubated with 10 µg/mL Hoechst 33,342 in the dark at 37.5 °C for seven minutes to mark the nuclei. Lastly, an inverted fluorescence microscope (Ti2eU; Nikon, Tokyo, Japan) and ImageJ version 8.0.2 software (NIH, Bethesda, MD, USA) were used to analyze the fluorescence intensities and to determine the numbers of total and apoptotic nuclei. We compared the level of apoptosis by calculating the percentage of apoptotic nuclei in the total number of blastocysts. The number of blastocysts on day 7 in the TUNEL staining assay was 15 to 20, and the assay was repeated more than three times.

### 2.5. 5-Ethynyl-2′-Deoxyuridine (EdU) Assay

The manufacturer’s instructions were followed to analyze cell proliferation using the BeyoClick EdU-555 Cell Proliferation Assay Kit (Beyotime, Shanghai, China). In short, on day 6 of the oocyte culture, 10% EdU was added to the IVC culture medium with or without CHE and then incubated in the dark at 38.5 °C in an atmosphere of 5% CO_2_ for 10 h. After incubation, the blastocysts developed to day 7 were washed four times with PBS-PVA, fixed in PBS-PVA containing 3.7% paraformaldehyde for 30 min, and then containing 0.1% Triton X-100 permeabilized for 30 min, all at room temperature. After permeabilization, the blastocysts were washed four times, mixed with 5% BeyoClick Additive Solution, and incubated in the dark at 38.5 °C in an atmosphere of 5% CO_2_ for 15 h. Finally, the blastocysts were incubated with 10 µg/mL Hoechst 33,342 in the dark at 37.5 °C for seven minutes to mark the nuclei. An inverted fluorescence microscope (Ti2eU; Nikon, Tokyo, Japan) and ImageJ version 8.0.2 software (NIH, Bethesda, MD, USA) were used to count the number of EdU-positive cells and total cells. We compared the level of cell proliferation by calculating the percentage of EdU-positive cells in the total number of blastocysts. The number of blastocysts on day 7 for the EDU staining assay was 15 to 20, and the assay was repeated more than three times.

### 2.6. Intracellular ROS and GSH Level Assay

We detected ROS and GSH levels using oocytes that had reached the 4-cell developmental stage. The oocytes were incubated in PBS-PVA, containing 10 µM 2′,7′-dichlorodihydrofluorescein diacetate (H2DCFDA, Beyotime, Shanghai, China) and 10 µM 4-chloromethyl-6,8-difluoro-7-hydroxycoumarin (CMF2HC, Beyotime, Shanghai, China), in the dark at 37.5 °C for 30 min. Next, we washed the oocytes four times in PBS-PVA and placed them into 3 µL of droplets of PBS-PVA. Finally, pictures were photographed using an inverted fluorescence microscope (Ti2eU; Nikon, Tokyo, Japan), and the fluorescence intensity was analyzed using ImageJ version 8.0.2 software (NIH, Bethesda, MD, USA). We collected 20 oocytes at the 4-cell stage of development each time for ROS and GSH level detection. Each indicator was tested over three replicates.

### 2.7. Mitochondrial Membrane Potential (MMP, ∆Ψ) Assay

The oocytes in the 4-cell stage are also used to detect the mitochondrial membrane potential (MMP); the oocytes were incubated in PBS-PVA containing 10 µg/mL 5,5’,6,6’-tetrachloro-1,1’,3,3’-tetraethylbenzi-midazolylcarbocyanineiodide iodide (JC-1, Beyotime, Shanghai, China) in the dark at 38.5 °C in an atmosphere of 5% CO_2_ for 17 h. After the oocytes were washed four times with PBS-PVA, the stained oocytes were put into 3 µL droplets of PBS-PVA. We used an inverted fluorescence microscope (Ti2eU; Nikon, Tokyo, Japan) to detect the red/green fluorescence signals and analyze the fluorescence intensities with ImageJ software. The average mitochondrial membrane potential (MMP) of the oocytes was calculated as the ratios of red fluorescence intensity (J-aggregates) to green fluorescence intensity (J-monomers). We tested MMP by collecting 10 to 15 oocytes at a time in the 4-cell developmental phase, and each group was repeated more than three times.

### 2.8. Immunofluorescence Staining

The day 7 blastocysts were collected and washed four times with PBS-PVA. The washed blastocysts were fixed in PBS-PVA containing 3.7% paraformaldehyde for 30 min and then in 0.1% Triton X-100 permeabilized for 30 min, all at room temperature. Next, the blastocysts were blocked with a blocking buffer (1% BSA in PBS-PVA) for one hour. After being blocked, the blastocysts were incubated with a primary LC3B antibody (1:200; Abcam, Cambridge, MA, USA; #ab48394) overnight in the dark at 4 °C. The blastocysts were washed four times the next day with PBS-PVA and then incubated with a secondary antibody (1:500, Cell Signaling; #8889, for LC3B staining) for one hour at 37.5 °C. After incubation, the oocytes were washed five times with PBS-PVA and incubated with 10 µg/mL Hoechst 33,342 for 10 min. Finally, the oocytes were washed five times with PBS-PVA and detected by an inverted fluorescence microscope (Ti2eU; Nikon, Tokyo, Japan). The level of autophagy in the oocytes was measured by the number of LC3B-positive dots. The number of blastocysts on day 7 for immunofluorescence staining was 15 to 20, and the test was repeated more than three times.

### 2.9. Quantitative Real-Time Reverse Transcription-Polymerase Chain Reaction (qRT-PCR)

Blastocysts on day 7 with similar size and quality were selected from the CHE groups (both with and without CHE; about 30 blastocysts were collected per group), and the extraction of total mRNA followed the manufacturer’s instructions, with the use of the Dynabeads™ mRNA DIRECT™ Purification Kit (Invitrogen). First-strand cDNA was obtained by reverse transcription using a reverse transcription kit (TIANGEN, Beijing, China). After reverse transcription, the concentration of cDNA was determined to ensure that the quality of the obtained cDNA was sufficient for subsequent qRT-PCR experiments. gDNA was removed in the reverse transcription process on the basis that the kit used contained heat-sensitive DNA digestion enzyme DNase I, which could digest gDNA at 37 °C, but with rapid inactivation in the reverse transcription reaction (55 °C or higher). The inactivated DNase I did not affect the subsequent cDNA, thereby excluding the interference of gDNA. According to the instructions, the KAPA SYBR FAST Qpcr Master Mix (2×) Kit (Kapa Biosystems, USA) was used to prepare qRT-PCR systems; each system was 20 µL, which contained 1 µL of cDNA template and 1 µL each of forward and reverse primers (the final primer concentration was 10 µM). The qRT-PCR process included initial denaturation for 30 min at 93 °C, and then 40 cycles were carried out; the conditions of each cycle were 3 s at 95 °C, 30 s at 60 °C and 20 s at 72 °C, and a final extension for 30 s at 37 °C. We included the melting curve analysis in the first few experiments and observed that it showed a single peak and was within a reasonable temperature range. The gene expression of two groups was analyzed by a LightCycler96, and the 2^−ΔΔCt^ method with GAPDH as the internal standard was used. The annealing temperature of all reactions was 60 °C. All primers used are shown in Table 1.

### 2.10. Statistical Analysis

The number of oocytes/blastocysts (n) used in each group and the number of repeated independent experiments (R) are shown in the figure note. Student’s t-test was used to calculate the pairwise statistical significance between two groups. One-way ANOVA (Tukey–Kramer) was used to compare the four means. The statistical analysis, including the checking of the normality of variables, was done using SPSS version 22.0 software (IBM Corp, Chicago, IL, USA), and the results are expressed as the mean ± standard deviation (SD). Results were considered significant as follows: * *p* < 0.05, ** *p* < 0.01, and *** *p* < 0.001.

## 3. Results

### 3.1. CHE Enhanced the Early Developmental Rates of the Porcine Embryos

**Figure 1:**
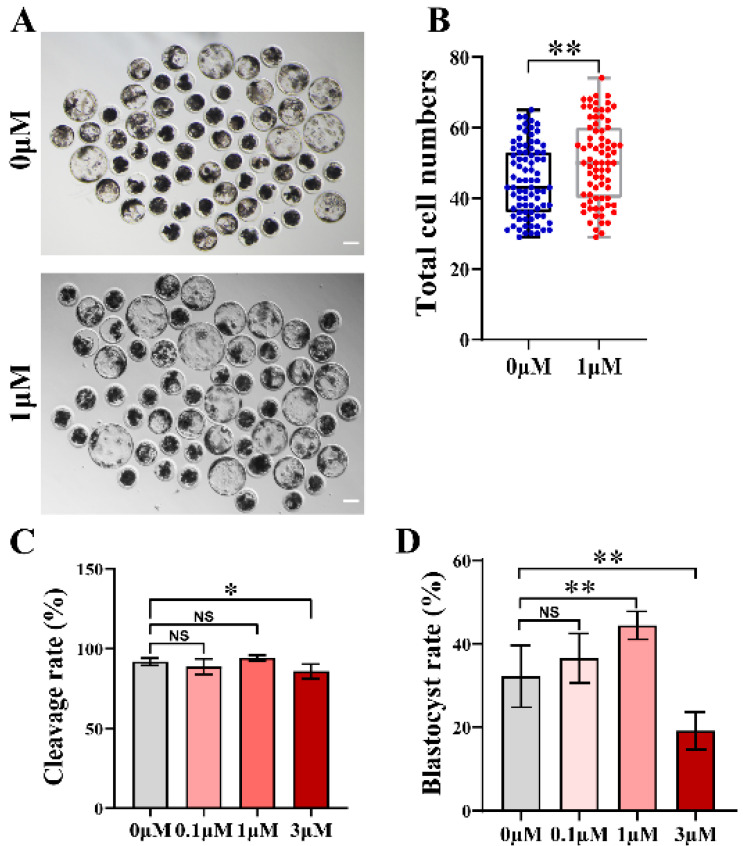
Effects of chrysoeriol (CHE) on the development of porcine parthenogenetic oocytes. (**A**) Embryo growth at 7 days in the 0 μM (control) group and 1 μM (CHE-treated) group. Scale bar = 100 μM. (**B**) Total number of cells in blastocysts on day 7 in the control group (n = 83) and the CHE-treated group (n = 75) R = 7. Data represent medians and maxima and minima. Significant difference is showed with ** (*p* < 0.01). (**C**) Cleavage rates of oocytes at different concentrations of CHE during development, divided into the 0 (n = 283), 0.1 (n = 282), 1 (n = 279), and 3 μM (n = 277) groups according to the concentration of CHE. R = 7. Differences are shown as NS (not significant) and significant, i.e., * (*p* < 0.05). (**D**) The blastocyst formation rate at 7 days at the different concentrations, 0 (n = 250), 0.1 (n = 249), 1 (n = 246), and 3 μM (n = 244). R = 6. Differences are NS (not significant) or significant, i.e., ** (*p* < 0.01).

In a preliminary experiment, we found no blastocyst formation at CHE concentrations of 5 or 10 μM in IVC medium, data not shown. Then, CHE at four concentrations (0, 0.1, 1, and 3 μM) was added to the IVC medium for formal experiments. The group with a concentration of 0 μM was defined as the control group. We found that the development of oocytes after CHE addition was better than that without CHE (Figure 1A). Parthenogenetically activated embryos can continue to grow and develop under electrical activation. Cleavage occurs to form parthenotes, so we tested the cleavage rate. After the addition of 0.1 and 1 μM CHE, there was no significant difference between the cleavage rate and the control group, but a decrease was observed in the 3 μM CHE group (Figure 1C). In terms of the blastocyst formation rate, a significant increase was observed in the 1 μM CHE treatment group, and 1 μM was determined to be the optimal treatment concentration for subsequent experiments. The CHE treatment of the CHE-treated group mentioned in the subsequent experiments is the 1 μM CHE group. (The blastocyst formation rates of the 0, 0.1, 1, and 3 μM groups were 35.15 ± 4.52, 28.39 ± 7.64, 44.53 ± 4.87, and 20.22 ± 3.99%, respectively; NS means not significant, *p* < 0.01) (Figure 1D). In addition, the total cell number of blastocysts in the 1 μM group (50.20 ± 11.35%) was higher than that in the control group (44.96 ± 10.11%, *p* < 0.01) (Figure 1B).

### 3.2. CHE Improved the Cell Proliferation Level and Reduced Apoptosis in Blastocysts

**Figure 2:**
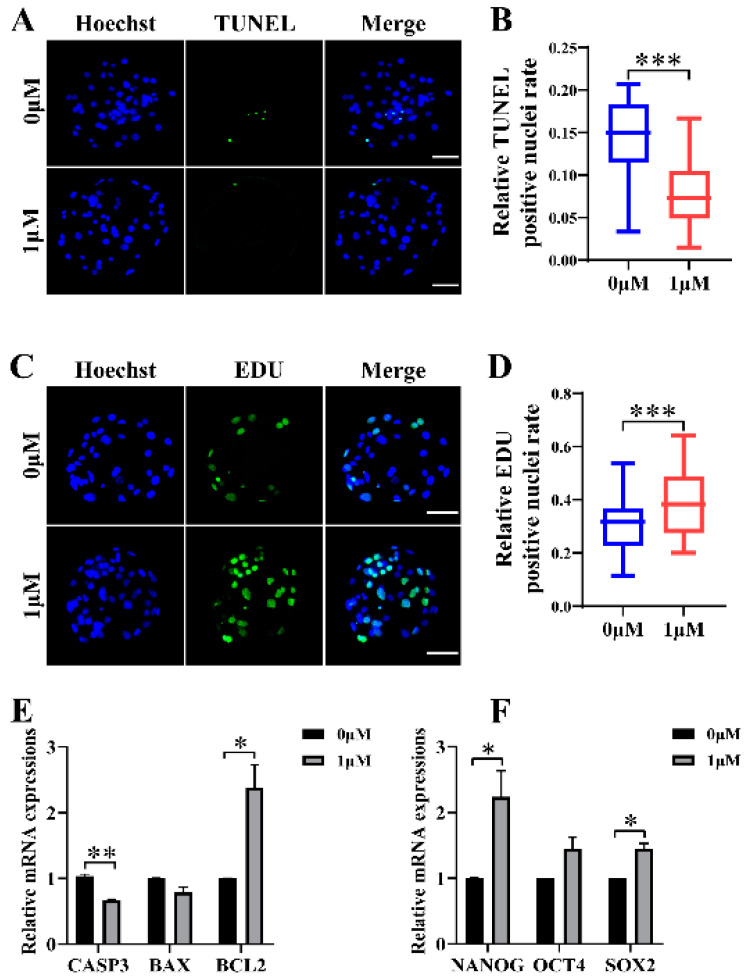
Effects of chrysoeriol (CHE) on cell proliferation and apoptosis. (**A**) TUNEL assay-dyed representative images of blastocysts. Scale bar = 50 μm. (**B**) Proportions of apoptotic nuclei in the control (0 μM) group (n = 56) and the CHE-treated (1 μM) group (n = 68) on day 7, R = 4. Significant differences are shown as *** (*p* < 0.001). (**C**) 5-ethynyl-2′-deoxyuridine (EdU)-dyed representative images of blastocysts. Scale bar = 50 μm. (**D**) Proportions of EdU-positive nuclei in the control (0 μM) group (n = 60) and the CHE-treated (1 μM) group (n = 60) on day 7, R = 5. Significant differences are shown as *** (*p* < 0.001). (**E**) Effect of CHE on the expression levels of apoptosis-related genes. Significant differences are shown as * (*p* < 0.05) and ** (*p* < 0.01). (**F**) Effect of CHE on the expression levels of cell proliferation-related genes. Significant differences are shown as * (*p* < 0.05).

To explore why the development rate of oocytes improved after 1 μM CHE treatment in the IVC stage, we first determined cell proliferation and apoptosis in blastocysts. The proportion of EdU-positive to total nuclei in the CHE-treated group (0.39 ± 0.12) was higher compared with the control group (0.30 ± 0.10, *p* < 0.001) (Figure 2C,D). We also measured the level of apoptosis in blastocysts with TUNEL assay, and the results showed the proportion of apoptotic nuclei to total nuclei in the CHE-treated group (0.07 ± 0.04) decreased compared with the control group (0.14 ± 0.05, *p* < 0.001) (Figure 2A,B). As expected, the qRT-PCR results showed a significant increase in the mRNA expression levels of genes associated with pluripotency and cell proliferation in the CHE-treated group, including SRY-box transcription factor 2 (*SOX2*) and Nanog homeobox (*NANOG*). Among the apoptosis-related genes, the mRNA expression levels of the anti-apoptotic gene B-cell lymphoma 2 (*BCL2*) increased significantly. At the same time, caspase 3 (*CASP3*) decreased in the CHE-treated group (Figure 2E,F).

### 3.3. CHE Improved the Antioxidant Capacity of Early-Stage Embryos

**Figure 3:**
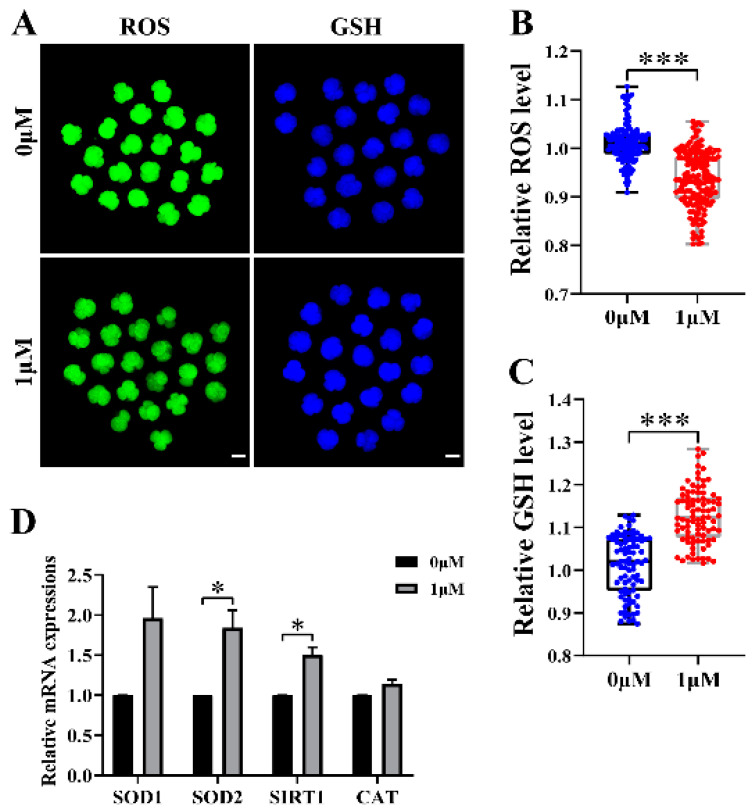
Effect of chrysoeriol (CHE) on the expression levels of ROS and GSH in embryos. (**A**) Representative pictures of H2DCFDA and CMF2HC staining in 4-cell stage embryos. Scale bar = 100 μM. (**B**) Relative fluorescence intensity changes of ROS in the control (0 μM) group (n = 137) and the CHE-treated (1 μM) group (n = 164) R = 5. Data represent medians and maxima and minima. Significant differences are shown as *** (*p* < 0.001). (**C**) Relative fluorescence intensity changes of GSH in the control (0 μM) group (n = 85) and the CHE-treated (1 μM) group (n = 85) R = 5. Data represent medians and maxima and minima. Significant differences are shown as *** (*p* < 0.001). (**D**) Changes in gene expression levels related to oxidative stress after the addition of CHE. Significant differences are shown as * (*p* < 0.05).

We chose to test reactive oxygen species (ROS) and glutathione (GSH) to explore whether CHE increased the antioxidant capacity during early embryonic development. Therefore, we detected the expression levels of ROS with 2′,7′-dichlorodihydrofluorescein diacetate (H2DCFDA) and of GSH with 4-chloromethyl-6,8-difluoro-7-hydroxycoumarin (CMF2HC) at the 4-cell stage of the porcine embryos (Figure 3A). The staining results showed that the relative level of ROS in the CHE-treated group was much lower than that in the control group (*p* < 0.001) (Figure 3B). At the same time, the relative level of GSH in the CHE-treated group was significantly higher than that in the control group (*p* < 0.001) (Figure 3C). As expected, the qRT-PCR results showed a significant increase in the mRNA expression levels of genes associated with oxidative stress in the CHE-treated group, including superoxide dismutase type 2 (*SOD2*) and sirtuin 1 (*SIRT1*) (Figure 3D).

### 3.4. CHE Reduces Autophagy Levels during Porcine Embryo Development

**Figure 4:**
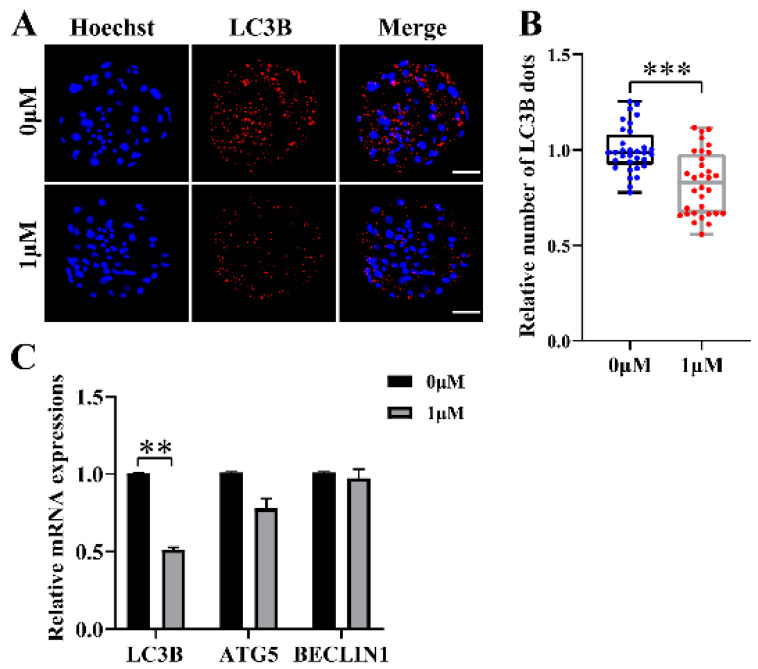
Effect of chrysoeriol (CHE) on the autophagy level of porcine embryos. (**A**) Hoechst and LC3B staining on day 7 in blastocysts. Scale bar = 50 μM. (**B**) Number of LC3B dots in the control (0 μM) (n = 32) and the CHE treatment (1 μM) (n = 32). R = 4. Data represent medians and maxima and minima. Significant differences are shown as *** (*p* < 0.001). (**C**) Changes in gene expression levels related to autophagy after the addition of CHE. Significant differences are shown as ** (*p* < 0.01).

To evaluate the effect on the level of autophagy in the embryo after the addition of CHE, blastocysts were stained, and the numbers of LC3B-positive spots was counted (Figure 4A), which were closely related to the autophagy level and are widely used to monitor autophagic activity. The results showed that the number of LC3B-positive dots in the CHE treatment group was significantly lower than that in the control group (*p* < 0.001) (Figure 4B). As expected, the qRT-PCR results showed a significant decrease in the mRNA expression levels of microtubule-associated protein 1 light chain 3 beta (*LC3B*) associated with autophagy in the CHE-treated group (Figure 4C).

### 3.5. CHE Improved the Mitochondrial Function of Early-Stage Porcine Embryos

**Figure 5:**
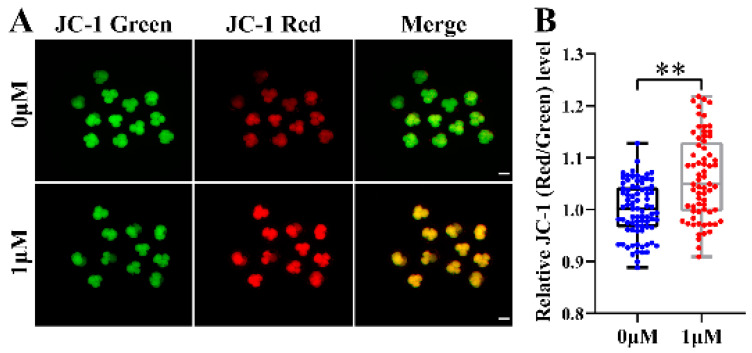
Effect of chrysoeriol (CHE) on the mitochondrial membrane potential (MMP). (**A**) Representative image of JC-1 staining at the 4-cell stage of porcine embryos. Scale bar = 100 μm. (**B**) Relative JC-1 red/green levels in the control (n = 79) and the CHE treatment (n = 68). R = 4. Data represent medians and maxima and minima. Significant differences are shown as ** (*p* < 0.01).

The mitochondrial membrane potential (MMP, Δ*Ψm*) is often used to measure the mitochondrial function and is an essential factor affecting embryonic development. We already showed that CHE supplementation increased cell proliferation and improved embryonic development. Therefore, to further explore other potential mechanisms of CHE, we assayed the MMP at the 4-cell stage of oocytes. In this experiment, the JC-1 stain was selected to detect MMP, and the evaluation index was the ratio of the fluorescence intensity of JC-1 in different colors (red/green). Representative images showed that MMP increased in the CHE treatment compared with the control group (*p* < 0.01) (Figure 5A,B). According to the results, CHE can up-regulate MMP to improve mitochondrial function and prevent mitochondrial damage and defects.

## 4. Discussion

The mature quality and subsequent developmental potential of embryos cultured in vitro are not as good as those in in vivo embryos due to the inability to achieve a perfect IVC medium and the difference in the cell’s external environment [33,34]. In addition, embryos are susceptible to ROS-induced oxidative stress during IVC culture [35], which can lead to stunted embryonic development and various early damage. We used parthenogenetically activated (PA) embryos in this study instead of IVF embryos. PA embryos have been widely accepted as a model for embryo development mechanism research because PA embryos have reasonable embryo development rates and can retain diploid characteristics. However, one limitation is that PA embryos are more sensitive to external oxidative stimuli, so the research results based on PA embryos may extend beyond the development of embryos produced by IVF.

Recently, research on antioxidants in food and compounds has become increasingly widespread. The previous research on the chemical chrysoeriol (CHE) mainly focused on its extraction from plants and its antioxidant activity. However, its effect on the reproductive system has yet to be reported. Thus, we speculate that CHE can improve embryo development due to its antioxidant effects.

In this experiment, parthenogenetic activation was used to simulate the in vivo fertilization process, and the oocytes were used to simulate the development process of the zygote in vitro. Parthenogenetically activated oocytes form cavities with inner cell masses and fluid when cultured for six to seven days and contain trophoblasts, called blastocysts [36]. Blastocysts formed on day 7 were selected for testing various indicators to evaluate the embryonic development capacity. The rate of blastocyst formation and the total number of cells in blastocysts increased when 1 μM CHE was added to the IVC medium. In addition, CHE treatment also improved cell proliferation and reduced the level of autophagy. These results show that CHE can improve the early embryonic development ability of porcine.

Porcine embryos also produce reactive oxygen species (ROS), and ROS include several types, including the hydroxyl radical (OH), radical (O^2−^), non-radical (H_2_O_2_), and organic peroxides [37]. To investigate possible mechanisms by which CHE enhances early embryonic development with antioxidant capacity, we detected ROS and GSH levels and MMP in the 4-cell stage of the embryos. In our study, the level of ROS in the CHE-treated group was significantly lower than that in the control group. CHE also reduced GSH levels and inhibited cellular endogenous ROS levels, consistent with the effect of supplementing other antioxidants.

Mitochondria can provide energy and perform other functions, playing an important role in the early stages of embryonic development [38]. Therefore, mitochondrial dysfunction impedes embryonic development and leads to apoptosis, autophagy, and even complete death [39]. MMP was detected after the addition of CHE, and an increase in MMP was observed, indicating that CHE protected mitochondria. Combined with the trend of the blastocyst rate, total cell number, and ROS and GSH levels, these results suggest that CHE can promote embryonic development and stabilize or even activate the mitochondrial function by reducing ROS.

Autophagy is necessary during early embryonic development, and defects can lead to developmental stagnation. Autophagy plays a vital role in the process from oocyte origin to fertilization. Recent findings have demonstrated that autophagy plays an essential role in the pathogenesis of polycystic ovary syndrome (PCOS), a prevalent disease among women of fertile age [40]. Therefore, the influences of autophagy in the normal control of embryonic development should be emphasized. Autophagy is also associated with the response to oxidative stress, redox signaling, cell death, and survival [41]. Therefore, we conducted a preliminary study on the changes in autophagy levels during the development of the porcine embryos. Our results suggest that the addition of CHE to the IVC medium reduced the autophagy levels, and this alteration was associated with changes in ROS levels and mitochondrial function in embryos.

In the qRT-PCR process, because of the stability of *GAPDH* and the slight change of *GAPDH* expression in different states, *GAPDH* was used as the internal reference in this experiment [42]. Lastly, we detected the mRNA expression of the oxidative stress-related genes, *SOD1*, *SOD2*, *SIRT1*, and *CAT*, and the pluripotency-related genes *NANOG*, *OCT4*, and *SOX2*, found them to be upregulated after CHE supplementation. These results indicate that the addition of CHE improved the embryo developmental competence and the ability of embryos to resist oxidative stress. On the contrary, the apoptosis-related genes, including *CASP3* and *BAX*, and autophagy-related genes, including *LC3B*, *ATG5*, and *BECLIN1*, decreased. Significantly, the anti-apoptosis gene *BCL2* was upregulated. The gene expression results show that CHE can reduce autophagy and apoptosis levels in porcine embryos.

We evaluated the effect of 1 µM CHE on enhancing the antioxidant capacity of porcine oocytes. To determine the antioxidant effect of CHE, ROS and GSH were tested, and then for a complete study and description of the role of CHE in porcine embryonic development, cell proliferation, apoptosis, and autophagy were also tested in this study. However, the research on apoptosis and autophagy is preliminary and does not involve the detection of protein expression, which is also a relatively difficult point in embryo research. In summary, this study is the first to report the antioxidant CHE, identify its potential to improve the development of porcine embryos, and preliminarily describe its mechanism. Whether CHE can also improve the embryo development of pigs and other animals in other ways, as well as its effect on different pathways, still needs further study.

## 5. Conclusions

In summary, we determined that at 1 µM, chrysoeriol (CHE) improved the early development of porcine embryos by increasing GSH levels and decreasing ROS levels. Furthermore, supplementation of CHE also reduced the proportion of apoptosis, enhanced mitochondrial function, and participated in regulating autophagy levels. These conclusions contribute to the improvement of in vitro culture and in vitro production of embryos.

## Figures and Tables

**Table 1:** Primer sequences used for qRT-PCR.

Genes	Sequences 5′–3′	Base
GAPDH	F: TTCCACGGCACAGTCAAG	18
	R: ATACTCAGCACCAGCATCG	19
SOD1	F: CAAAGGATCAAGAGAGGCACG	21
	R: CGAGAGGGCGATCACAGAAT	20
SOD2	F: TTCTGGACAAATCTGAGCCCTAACG	25
	R: CGACGGATACAGCGGTCAACTTC	23
SIRT1	F: GAGAAGGAAACAATGGGCCG	20
	R: ACCAAACAGAAGGTTATCTCGGT	23
CAT	F: AACTGTCCCTTCCGTGCTA	19
	R: CCTGGGTGACATTATCTTCG	20
NANOG	F: TGTCTCTCCTCTTCCTTCCTCCATG	25
	R: TCCTCCTTCTCTGTGCTCTTCTCTG	25
OCT4	F: CCTATGACTTCTGCGGAGGGA	21
	R: TTTGATGTCCTGGGACTCCTCG	22
SOX2	F: GAACAGCCCAGACCGAGTTAAGC	23
	R: CTGATCTCCGAGTTGTGCATCTTGG	25
CASP3	F: AGAATTGGACTGTGGGATTGAGACG	25
	R: GCCAGGAATAGTAACCAGGTGCTG	24
BAX	F: GGACTTCCTTCGAGATCGGC	20
	R: GCGTCCCAAAGTAGGAGAGG	20
BCL2	F: GGATAACGGAGGCTGGGATG	20
	R: TTATGGCCCAGATAGGCACC	20
LC3B	F: TTCAAACAGCGCCGAACCTT	20
	R: TTTGGTAGGATGCTGCTCTCG	21
ATG5	F: TTGCAGTGGCTGAGTGAACA	20
	R: TCAATCTGTTGGTTGCGGGA	20
BECLIN1	F: GATGGTGGCTTTCCTGGACTGTG	23
	R: ACTGCCTCCTGTGTCTTCAATCTTG	25

F: forward primer; R: reverse primer; GAPDH: glyceraldehyde 3-phosphate dehydrogenase; SOD1: superoxide dismutase type 1; SOD2: superoxide dismutase type 2; SIRT1: sirtuin 1; CAT: catalase; NANOG: Nanog homeobox; OCT4: octamer-binding transcription factor 4; SOX2: RY-box transcription factor 2; CASP3: caspase 3; BAX: Bcl-2 associated X-protein; BCL2: B-cell lymphoma 2; LC3B: microtubule associated protein 1 light chain 3 beta; ATG5: autophagy-related 5.

## Data Availability

The data presented in this study are available on request from the corresponding author.
